# Tracking of time-varying genomic regulatory networks with a LASSO-Kalman smoother

**DOI:** 10.1186/1687-4153-2014-3

**Published:** 2014-02-12

**Authors:** Jehandad Khan, Nidhal Bouaynaya, Hassan M Fathallah-Shaykh

**Affiliations:** 1 Department of Electrical and Computer Engineering, Rowan University, 201 Mullica Hill Rd, Glassboro, NJ 08028, USA; 2 Department of Neurology, University of Alabama at Birmingham, 1720 2nd Ave S, Birmingham, AL 35233, USA; 3 Department of Mathematics, University of Alabama at Birmingham, 1720 2nd Ave S, Birmingham, AL 35233, USA; 4 Department of Electrical and Computer Engineering, University of Alabama at Birmingham, 1720 2nd Ave S, Birmingham, AL 35233, USA; 5 Department of Biomedical Engineering, University of Alabama at Birmingham, 1720 2nd Ave S, Birmingham, AL 35233, USA; 6 Department of Cell, Developmental and Integrative Biology, University of Alabama at Birmingham, 1720 2nd Ave S, Birmingham, AL 35233, USA

## Abstract

It is widely accepted that cellular requirements and environmental conditions dictate the architecture of genetic regulatory networks. Nonetheless, the status quo in regulatory network modeling and analysis assumes an invariant network topology over time. In this paper, we refocus on a dynamic perspective of genetic networks, one that can uncover substantial topological changes in network structure during biological processes such as developmental growth. We propose a novel outlook on the inference of time-varying genetic networks, from a limited number of noisy observations, by formulating the network estimation as a target tracking problem. We overcome the limited number of observations (small *n* large *p* problem) by performing tracking in a compressed domain. Assuming linear dynamics, we derive the LASSO-Kalman smoother, which recursively computes the minimum mean-square sparse estimate of the network connectivity at each time point. The LASSO operator, motivated by the sparsity of the genetic regulatory networks, allows simultaneous signal recovery and compression, thereby reducing the amount of required observations. The smoothing improves the estimation by incorporating all observations. We track the time-varying networks during the life cycle of the *Drosophila melanogaster*. The recovered networks show that few genes are permanent, whereas most are transient, acting only during specific developmental phases of the organism.

## 1 Introduction

### 1.1 Motivation

A major challenge in systems biology today is to understand the behaviors of living cells from the dynamics of complex genomic regulatory networks. It is no more possible to understand the cellular function from an informational point of view without unraveling the underlying regulatory networks than to understand protein binding without knowing the protein synthesis process. The advances in experimental technology have sparked the development of genomic network inference methods, also called *reverse engineering* of genomic networks. Most popular methods include (probabilistic) Boolean networks [1,2], (dynamic) Bayesian networks [3-5], information theoretic approaches [6-9], and differential equation models [10-12]. A comparative study is compiled in [13]. The Dialogue on Reverse Engineering Assessment and Methods (DREAM) project, which built a blind framework for performance assessment of methods for gene network inference, showed that there is no single inference method that performs optimally across all data sets. In contrast, integration of predictions from multiple inference methods shows robust and high performance across diverse data sets [14].

These methods, however, estimate one single network from the available data, independently of the cellular ‘themes’ or environmental conditions under which the measurements were collected. In signal processing, it is senseless to find the Fourier spectrum of a non-stationary time series [15]. Similarly, time-dependent genetic data from dynamic biological processes such as cancer progression, therapeutic responses, and developmental processes cannot be used to describe a unique time-invariant or static network [16,17]. Inter- and intracellular spatial cues affect the course of events in these processes by rewiring the connectivity between the molecules to respond to specific cellular requirements, e.g., going through the successive morphological stages during development. Inferring a unique static network from a time-dependent dynamic biological process results in an ‘average’ network that cannot reveal the regime-specific and key transient interactions that cause cell biological changes to occur. For a long time, it has been clear that the evolution of the cell function occurs by change in the genomic program of the cell, and it is now clear that we need to consider this in terms of change in regulatory networks [16,17].

### 1.2 Related work

While there is a rich literature on modeling static or time-invariant networks, much less has been done towards inference and learning techniques for recovering topologically rewiring networks. In 2004, Luscombe et al. made the earliest attempt to unravel topological changes in genetic networks during a temporal cellular process or in response to diverse stimuli [17]. They showed that under different cellular conditions, transcription factors, in a genomic regulatory network of *Saccharomyces cerevisiae*, alter their interactions to varying degrees, thereby rewiring the network. Their method, however, is still based on a static representation of known regulatory interactions. To get a dynamic perspective, they integrated gene expression data for five conditions: cell cycle, sporulation, diauxic shift, DAN damage, and stress response. From these data, they traced paths in the regulatory network that are active in each condition using a trace-back algorithm [17].

The main challenge facing the community in the inference of time-varying genomic networks is the unavailability of multiple measurements of the networks or multiple observations at every instant *t*. Usually, one or at most a few observations are available at each instant. This leads to the ‘large *p* small *n*’ problem, where the number of unknowns is smaller than the number of available observations. The problem may seem ill defined because no unique solution exists. However, we will show that this hurdle can be circumvented by using prior information.

One way to ameliorate this data scarcity problem is to presegment the time series into stationary epochs and infer a static network for each epoch separately [18,18-23]. The segmentation of the time series into stationary pieces can be achieved using several methods including estimation of the posterior distribution of the change points [19], HMMs [20], clustering [18], detecting geometric structures transformed from time series [21], and MCMC sampling algorithm to learn the times of non-stationarities (transition times) [22,23]. The main problem with the segmentation approach for estimating time-varying gene networks is the limited number of time points available in each stationary segment, which is a subset of the already limited data. Since the time-invariant networks are inferred in each segment using only the data points within that segment and disregarding the rest of the data, the resulting networks are limited in terms of their temporal resolution and statistical power.

A semi-flexible model based on a piecewise homogeneous dynamic Bayesian network, where the network structure in each segment shares information with adjacent segments, was proposed in [24]. This setting allows the network to vary gradually through segments. However, some information is lost by not considering the entire data samples for the piecewise inference. A more flexible model of time-varying Bayesian networks based on a non-parametric Bayesian method for regression was recently proposed in [25]. The non-parametric regression is expected to enable capturing of non-linear dynamics among genes [24]. However, a full-scale study of a time-varying system was lacking; the approach was only tested on an 11-gene *Drosophila melanogaster* network.

Full resolution techniques, which allow a time-specific network topology to be inferred from samples measured over the entire time series, rely on model-based approaches [26,27]. However, these methods learn the structure (or skeleton) of the network, but not the detailed strength of the interactions between the nodes. Dynamic Bayesian networks (DBNs) have been extended to the time-varying case [28-31]. Among the earliest models is the time-varying autoregressive (TVAR) model [29], which describes nonstationary linear dynamic systems with continuously changing linear coefficients. The regression parameters are estimated recursively using a normalized least-squares algorithm. In time-varying DBNs (TVDBN), the time-varying structure and parameters of the networks are treated as additional hidden nodes in the graph model [28].

In summary, the current state-of-the-art in time-varying network inference relies on either chopping the time-series sequence into homogeneous subsequences [18-23,32-35] (concatenation of static networks) or extending graphical models to the time-varying case [28-31] (time modulation of static networks).

### 1.3 Proposed work and contributions

In this paper, we propose a novel formulation of the inference of time-varying genomic regulatory networks as a tracking problem, where the target is a set of incoming edges for a given gene. We show that the tracking can be performed in parallel: there are *p* independent trackers, one for each gene in the network, thus avoiding the curse of dimensionality problem and reducing the computation time. Assuming linear dynamics, we use a constrained and smoothed Kalman filter to track the network connections over time. At each time instant, the connections are characterized by their strength and sign, i.e., stimulative or inhibitive. The sparsity constraint allows simultaneous signal recovery and compression, thereby reducing the amount of required observations. The smoothing improves the estimation by incorporating all observations for each smoothed estimate. The paper is organized as follows: In Section 2, we formulate the network inference problem in a state-space framework, where the target state, at each time point, is the network connectivity vector. Assuming linear dynamics of gene expressions, we further show that the model can be decomposed into *p* independent linear models, *p* being the number of genes. Section 3 derives the LASSO-Kalman smoother, which renders the optimal network connectivity at each time point. The performance of the algorithm is assessed using synthetic data in Section 4. The LASSO-Kalman smoother is subsequently used to recover the time-varying networks of the *D. melanogaster* during the time course of its development spanning the embryonic, larval, pupal, and adulthood periods.

## 2 The state-space model

Static gene networks have been modeled using a standard state-space representation, where the state ***x***_*k*_ represents the gene expression values at a particular time *k*, and the microarray data ***y***_*k*_ constitutes the set of noisy observations [36,37]. A naive approach to tackle the time-varying inference problem is to generalize this representation of time-invariant networks and augment the gene profile state vector by the network parameters at all time instants. This approach, however, will result in a very poor estimate due to the large number of unknown parameters. Instead, we propose to re-formulate the state-space model as a function of the time-varying connections or parameters rather than the gene expression values. In order to do so, we need to model the time evolution of the parameters using, for instance, prior knowledge about the biological process. Denoting by ***a***_*k*_ the network parameters to be estimated, the state-space model of the time-varying network parameters can be written as

(1) $$a(k+1)=f_{k}(a(k))+w(k),$$

(2) $$y(k)=g_{k}(a(k))+v(k).$$

The function *f*_*k*_ models the dynamical evolution of the network parameters, e.g., smooth evolution or abrupt changes across time. The observation function *g*_*k*_ characterizes the regulatory relationships among the genes and can be, for instance, derived from a differential equation model of gene expression (see Equation 8). In particular, observe that the state-space model in (1) to (2) does not incorporate the ‘true’ gene expression values, which have to be estimated and subsequently discarded. It only includes the measured gene expression values with an appropriate measurement noise term.

### 2.1 The observation model

We model the concentrations of mRNAs, proteins, and other molecules using a time-varying ordinary differential equation (ODE). More specifically, the concentration of each molecule is modeled as a linear function of the concentrations of the other components in the system. The time-dependent coefficients of the linear ODE capture the rewiring structure of the network. We have

(3) $${\dot{x}}_{i}(t)=-\lambda_{i}(t)x_{i}(t)+\overset{p}{\underset{j=1}{\sum }}w_{\text{ij}}(t)x_{j}(t)+b_{i}u(t)+v_{i}(t),$$

where *i*=1,⋯,*p*, *p* being the number of genes, *x*_*i*_(*t*) is the expression level of gene *i* at time *t*, ${\dot{x}}_{i}(t)$ is the rate of change of expression of gene *i* at time *t*, *λ*_*i*_ is the self degradation rate, *w*_*ij*_(*t*) represents the time-varying influence of gene *j* on gene *i*, *b*_*i*_ is the effect of the external perturbation *u*(*t*) on gene *i*, and *v*_*i*_(*t*) models the measurement and biological noise. The goal is to infer the time-varying gene interactions $\lambda_{i}(t),{\left\{w_{\text{ij}}(t)\right\}}_{i,j=1}^{p}$, given a limited number of measurements *n*<*p*.

To simplify the notation, we absorb the self-degradation rate *λ*_*i*_(*t*) into the interaction parameters by letting *a*_*ij*_(*t*)=*w*_*ij*_(*t*)-*λ*_*i*_(*t*)*δ*_*ij*_, where *δ*_*ij*_ is the Kronecker delta function. The external perturbation is assumed to be known. The model in (3) can be simplified by introducing a new variable

(4) $$y_{i}(t)={\dot{x}}_{i}(t)-b_{i}u(t).$$

The discrete-time equivalent of (3) can, therefore, be expressed as

(5) $$y_{i}(k)=\;\overset{p}{\underset{j=1}{\sum }}a_{\text{ij}}(k)x_{j}(k)+v_{i}(k),\;i=1,\cdots \;,p,\;k=1,\ldots ,\mathrm{n.}$$

Writing (5) in matrix form, we obtain

(6) y(k)=A(k)x(k)+v(k),

where **y**(*k*)= [*y*_1_(*k*),…,*y*_*p*_(*k*)]^*T*^, *A*(*k*)={*a*_*ij*_(*k*)} is the matrix of time-dependent interactions, **x**(*k*)=[*x*_1_(*k*),…*x*_*p*_(*k*)]^*T*^, and **v**(*k*)=[*v*_1_(*k*),…,*v*_*p*_(*k*)]^*T*^.

Let 1≤*m*_*k*_<*p* be the number of available observations at time *k*. Taking into account all *m*_*k*_ observations, Equation 6 becomes

(7) Y(k)=A(k)X(k)+V(k),

where ***Y***(*k*),***X***(*k*), and $V(k)\in R^{p\times m_{k}}$ with the *m*_*k*_ observations ordered in the columns of the corresponding matrices.

The linear model in Equation 7 can be decomposed into *p* independent linear models as follows:

(8) $$\begin{array}{l} y_{i}^{t}(k)=a_{i}^{t}(k)X(k)+v_{i}^{t}(k), \end{array}$$

where $y_{i}^{t}(k),a_{i}^{t}(k)$, and $v_{i}^{t}(k)$ are the *i*th rows of ***Y***(*k*),***A***(*k*), and ***V***(*k*), respectively. In particular, the vector ***a***_*i*_(*k*) represents the set of incoming edges to gene *i* at time *k*. Equation 8 represents the observation equation for gene *i*.

### 2.2 The linear state-space model

**Figure 1:**
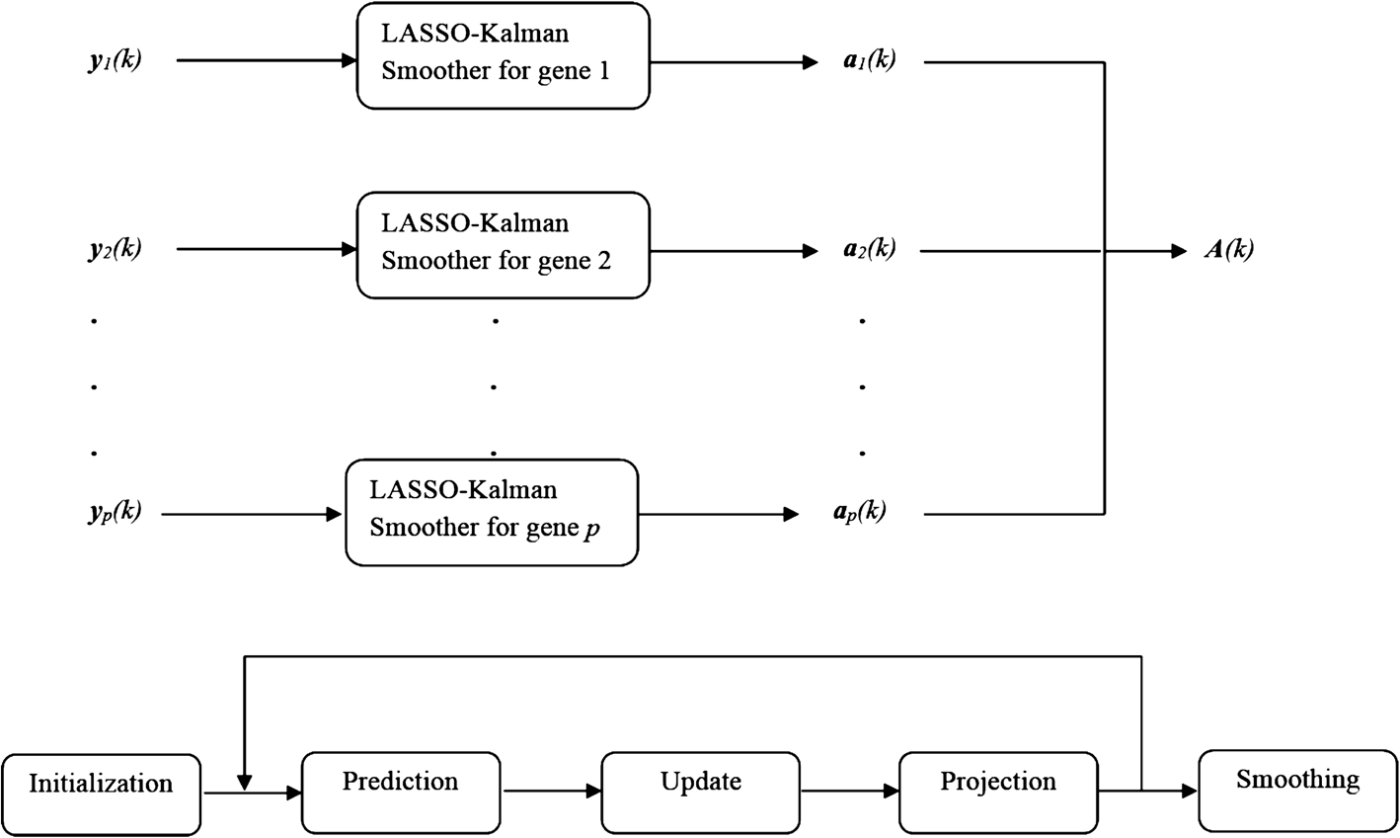
**The LASSO-Kalman smoother tracker.** Top row: parallel architecture of the tracker. The tracking is performed for each gene separately to find its incoming edges. The connectivity matrix $A(k)=[a_{1}^{t};\cdots \;;a_{p}^{t}]$. Bottom row: the LASSO-Kalman smoother: the prior estimate is predicted to give ***a***_*k*|*k*-1_. The filter is updated with the observations to give the unconstrained estimate ***a***_*k*|*k*_. The projection operator projects this estimate to enforce the constraint. This procedure is repeated for all time steps *k*=1,⋯,*n*. Then, a forward-backward smoother is applied to reduce the covariance of the estimate and lead to the final constrained and smoothed estimate.

The state equation models the dynamics of the state vector ***a***_*i*_(*k*) given *a priori* knowledge of the system. In this work, we assume a random walk model of the network parameters. The random walk model is chosen for two reasons. First, it reflects a flat prior or a lack of *a priori* knowledge. Second, it leads to a smooth evolution of the state vector over time (if the variance of the random walk is not very high). The state space model of the incoming edges for gene *i* is, therefore, given by

(9) $$\left\{\begin{array}{l} a_{i}(k+1)=a_{i}(k)+w_{i}(k)\\ y_{i}(k)=X^{t}(k)a_{i}(k)+v_{i}(k), \end{array}\right)$$

where *i*=1,⋯,*p*, and ***w***_*i*_(*k*) and ***v***_*i*_(*k*) are, respectively, the process noise and the observation noise, assumed to be zero mean Gaussian noise processes with known covariance matrices, *Q*(*k*) and *R*(*k*), respectively. In addition, the process and observation noises are assumed to be uncorrelated with each other and with the state vector ***a***_*i*_(*k*). In particular, we have *p* independent state-space models of the form (9) for *i*=1,⋯,*p*. Thus, the connectivity matrix *A* can be recovered by simultaneous recovery of its rows. Another important advantage of the representation in (9) is that the state vector ***a***_*i*_(*k*) has dimension *p* (the number of genes in the network) rather than *p*^2^ (the number of possible connections in the network), thus avoiding the curse of dimensionality problem. For instance, in a network of 100 genes, the state vector will have dimension 100 instead of 10,000!. Though the number of genes *p* can be large, we show in simulations that the performance of the Kalman tracker is unchanged for *p* as large as 5,000 genes by using efficient matrix decompositions to find the numerical inverse of matrices of size *p*. A graphical representation of the parallel architecture of the tracker is shown in Figure 1.

It is well known that the minimum mean square estimator, which minimizes $E[∥a(k)-\hat{a}(k){∥}_{2}^{2}]$, can be obtained using the Kalman filter if the system is observable. If the system is unobservable, then the classical Kalman filter cannot recover the optimal estimate. In particular, it seems hopeless to recover $a_{i}(k)\in R^{p}$ in (9) from an under-determined system where *m*_*k*_<*p*. Fortunately, this problem can be circumvented by taking into account the fact that ***a***_*i*_(*k*) is sparse. Genomic regulatory networks are known to be sparse: each gene is governed by only a small number of the genes in the network [11].

## 3 The LASSO-Kalman smoother

### 3.1 Sparse signal recovery

Recent studies [38,39] have shown that sparse signals can be exactly recovered from an under-determined system of linear equations by solving the optimization problem

(10) $$\min∥\hat{z}{∥}_{0}\;\text{s.t.}\;∥y-H\hat{z}{∥}_{2}^{2}\leq \epsilon ,$$

for a sufficiently small *ϵ* and where the *l*_0_-norm, ∥***z***∥_0_, denotes the support of ***z*** or the number of non-zero elements in ***z***. The optimization problem in (10) can be extended to the stochastic case as follows:

(11) $$\min∥\hat{z}{∥}_{0}\;\text{s.t.}\;E_{z|y}[\;∥z-\hat{z}{∥}_{2}^{2}]\leq \mathrm{\epsilon .}$$

Unfortunately, the above optimization problem is, in general, NP-hard. However, it has been shown that if the observation matrix ***H*** obeys the restricted isometry property (RIP), then the solution of the combinatorial problem (10) can be recovered by solving instead the convex optimization problem

(12) $$\min∥\hat{z}{∥}_{1}\;\text{s.t.}\;∥y-H\hat{z}{∥}_{2}^{2}\leq \mathrm{\epsilon .}$$

This is a fundamental result in the emerging theory of *compressed sensing*(CS) [38,39]. CS reconstructs large dimensional signals from a small number of measurements, as long as the original signal is sparse or admits a sparse representation in a certain basis. Compressed sensing has been implemented in many applications including digital tomography [38], wireless communication [40], image processing [41], and camera design [42]. For a further review of CS, the reader can refer to [38,39].

Inspired by the compressed sensing approach given that genomic regulatory networks are sparse, we formulate a constrained Kalman objective

(13) $$\underset{\hat{z}}{\min}E_{z|y}\left[∥z-\hat{z}{∥}_{2}^{2}\right]\;\text{s.t.}\;∥\hat{z}{∥}_{1}\leq \mathrm{\epsilon .}$$

The constrained Kalman objective in (13) can be seen as the regularized version of least squares known as least absolute shrinkage and selection operator (LASSO) [43], which uses the *l*_1_ constraint to prefer solutions with fewer non-zero parameter values, effectively reducing the number of variables upon which the given solution is dependent. For this reason, the LASSO and its variants are fundamental to the theory of compressed sensing.

### 3.2 Constrained Kalman filtering

Constrained Kalman filtering has been mainly investigated in the case of linear equality constraints of the form ***Dx***=***d***, where ***D*** is a known matrix and ***d*** is a known vector [44]. The most straightforward method to handle linear equality constraints is to reduce the system model parametrization [45]. This approach, however, can only be used for linear equality constraints and cannot be used for inequality constraints (i.e., constraints of the form ***Dx***≤***d***). Another approach is to treat the state constraints as perfect measurements or pseudo-observations (i.e., no measurement noise) [46]. The perfect measurement technique applies only to equality constraints as it augments the measurement equation with the constraints. The third approach is to project the standard (unconstrained) Kalman filter estimate onto the constraint surface [44]. Though non-linear constraints can be linearized and then treated as perfect observations, linearization errors can prevent the estimate from converging to the true value. Non-linear constraints are, thus, much harder to handle than linear constraints because they embody two sources of errors: truncation errors and base point errors [47,48]. Truncation errors arise from the lower order Taylor series approximation of the constraint, whereas base point errors are due to the fact that the filter linearizes around the estimated value of the state rather than the true value. In order to deal with these errors, iterative steps were deemed necessary to improve the convergence towards the true state and better enforce the constraint [47-49]. The number of necessary iterations is a tradeoff between estimation accuracy and computational complexity.

In this work, the non-linear constraint is the *l*_1_-norm of the state vector. We adopt the projection approach, which projects the unconstrained Kalman estimate at each step onto the set of sparse vectors, as defined by the constraint in (13). Denoting by $\tilde{a}$ the unconstrained Kalman estimate, the constrained estimated, $\hat{a}$, is then obtained by solving the following (convex) LASSO optimization:

(14) $$\hat{a}=\underset{a}{\text{argmin}}\;∥\tilde{a}-a{∥}_{2}^{2}+\lambda ∥a{∥}_{1},$$

where *λ* is a parameter controlling the tradeoff between the residual error and the sparsity. This approach is motivated by two reasons: First, we found through extensive simulations that the projection approach leads to more accurate estimates than the iterative pseudo-measurement techniques (PM) in [47-49]. Additionally, the sparsity constraint is controlled by only one parameter, namely *λ*, whereas in PM, the number of iterations is a second parameter that needs to be properly tuned and presents a tradeoff between accuracy and computational time. Second, for large-scale genomic regulatory networks (few thousands of genes), the iterative PM approaches render the constrained Kalman tracking problem computationally prohibitive.

### 3.3 The LASSO-Kalman smoother

The Kalman filter is causal, i.e., the optimal estimate at time *k* depends only on past observations {***y***(*i*),*i*≤*k*. In the case of genomic measurements, all observations are recorded and available for post-processing. By using all available measurements, the covariance of the optimal estimate can be reduced, thus improving the accuracy. This is achieved by smoothing the Kalman filter using a forward-backward approach [44]. The forward-backward approach obtains two estimates of ***a***(*j*). The first estimate, ${\hat{a}}_{f}$, is based on the standard Kalman filter that operates from *k*=1 to *k*=*j*. The second estimate, ${\hat{a}}_{b}$, is based on a Kalman filter that runs backward in time from *k*=*n* back to *k*=*j*. The forward-backward approach combines the two estimates to form an optimal smoothed estimate. The LASSO-Kalman smoother algorithm is summarized below (see also Figure 1).

#### Algorithm 1 **The LASSO-Kalman smoother algorithm**

## 4 Results and discussion

### 4.1 Synthetic data

In order to assess the efficacy of the proposed LASSO-Kalman smoother in estimating the connectivity of time-varying networks, we first perform Monte Carlo simulations on the generated data to assess the prediction error using the following criterion:

(23) $$∥a_{\text{ij}}-\hat{a_{\text{ij}}}∥\leq \alpha |a_{\text{ij}}|$$

where *a*_*ij*_ is the (*i*,*j*)th true edge value and $\hat{a_{\text{ij}}}$ is the corresponding predicted edge value. The criterion in (15) counts an error if the estimated edge value is outside an *α*-vicinity of the true edge value. In our simulations, we adopted a value of *α* equal to 0.2. That is, the error tolerance interval is ± 20*%* of the true value. The percentage of total correct or incorrect edges in a connectivity matrix is used to determine the accuracy of the algorithm.

**Figure 2:**
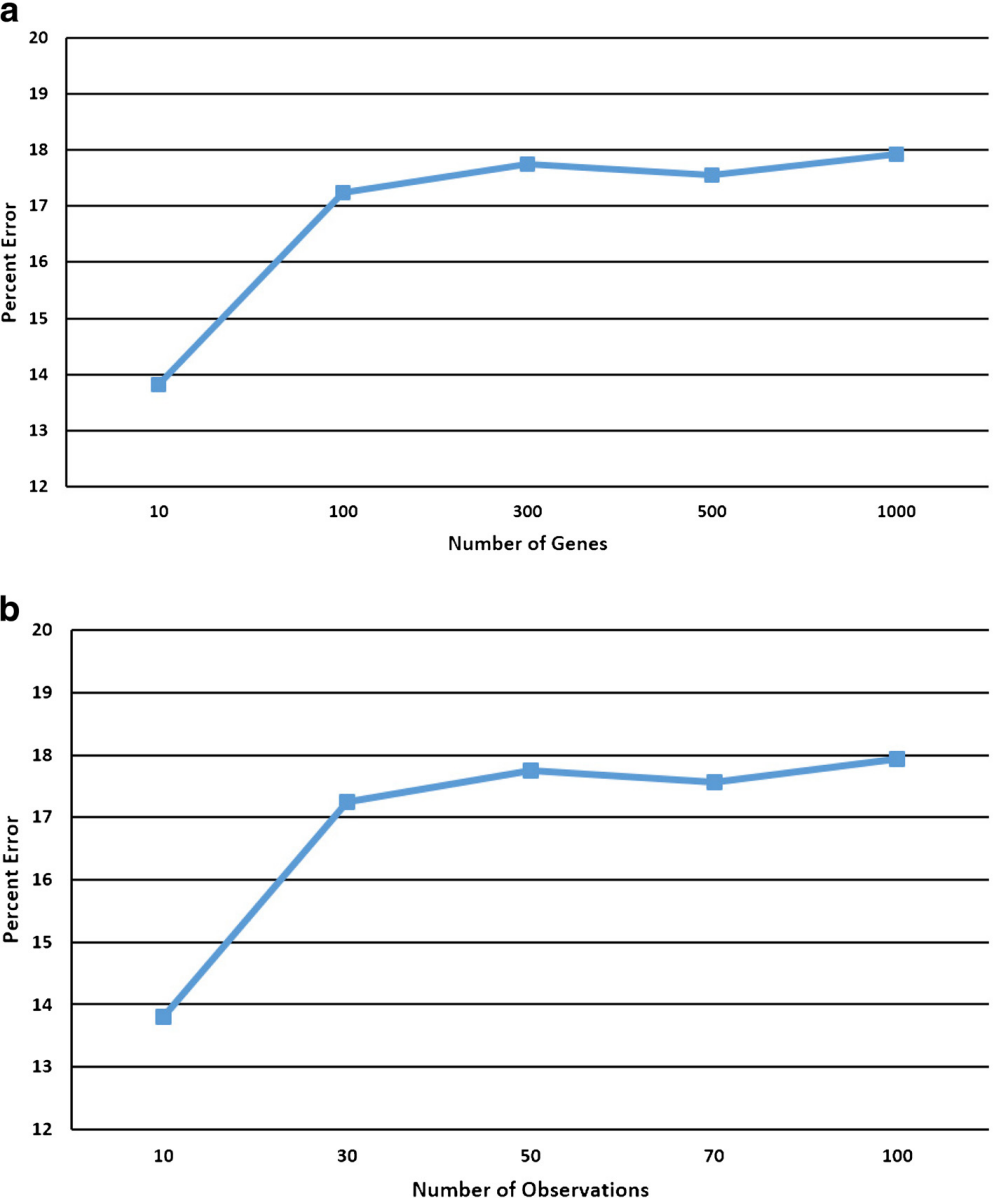
**Effect of network size and number of observations on the prediction error.(a)** Effect of the network size on the prediction error. **(b)** Effect of the number of observations on the prediction error.

We first investigate the effect of the network size on the estimation error. We generate networks of different sizes according to the model in (7) and calculate the prediction error. Figure 2a shows the prediction error as a function of the network size with a number of measurements equal to 70% of the network size *p*. We observe that the network estimation error is about constant between *p*=100 to *p*=1,000 and is thus unaffected by how large the network is, at least for networks of size few thousand genes. The reason for this outcome may be the linear increase of the size vector with the number of genes, which is due to the splitting of the original connectivity estimation problem (*p*^2^ parameters) into *p* smaller problems, that can be solved simultaneously.

**Figure 3:**
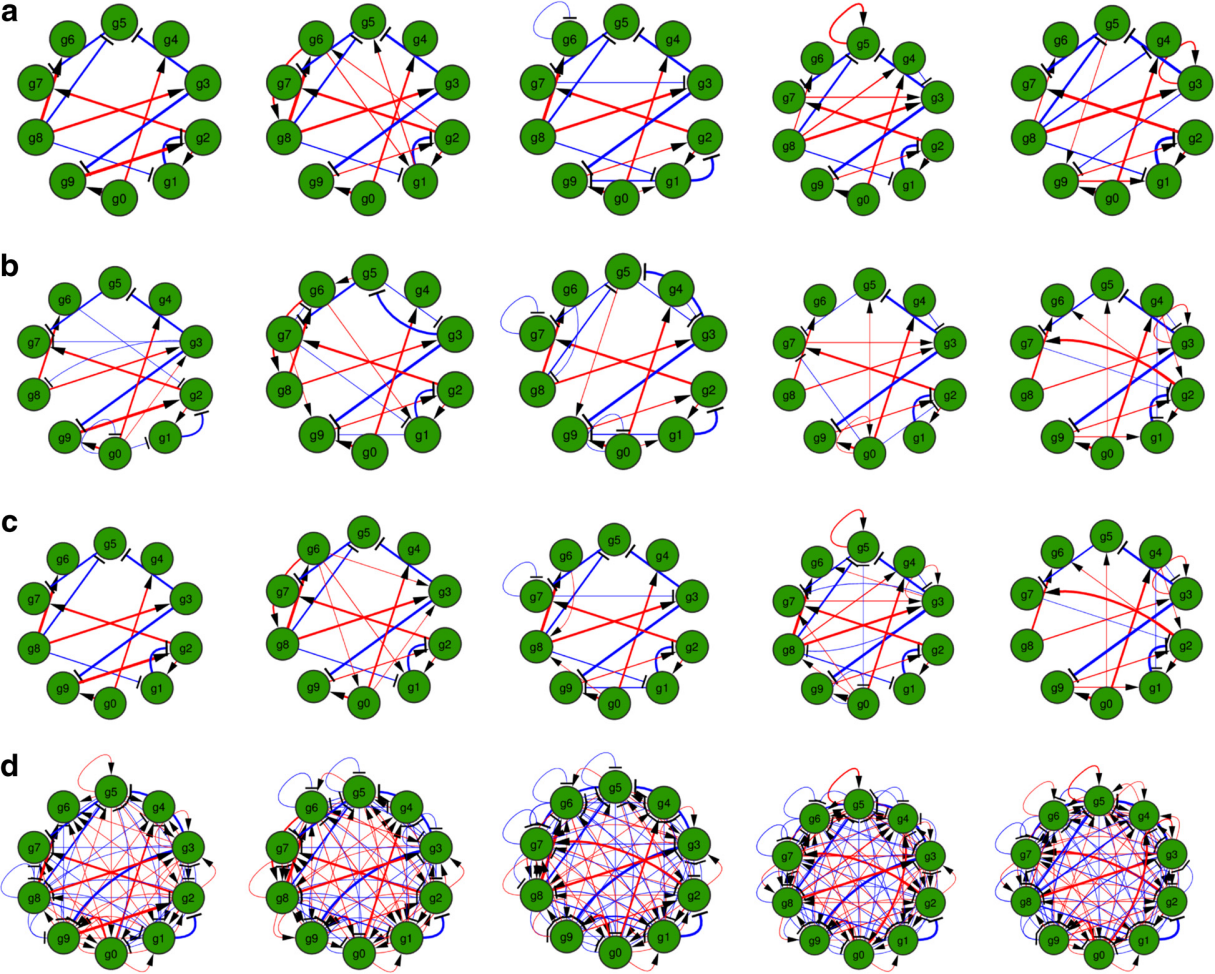
**Tracking of a ten-gene network.** The network evolved over five time points, with seven observations or measurements available at each time point. **(a)** Time-varying true network evolving over five time points, with seven observations available per time point. **(b)** Estimated time-varying network using the LASSO-Kalman smoother. **(c)** Estimated time-varying network using the LASSO-Kalman filter (no smoothing). **(d)** Estimated time-varying network using the classical Kalman filter.

We subsequently investigated the effect of the number of measurements *m* on the prediction accuracy. Figure 2b shows the prediction error as a function of the number of observations for a network of size *p*=100. The estimation error seems to be constant up to 50 measurements then decreases rapidly as the number of observations increase to 100. But even for a small number of observations (10% of the network size), the estimation error is fairly small (less than 18%). This is an important result because in real-world applications, the number of available observations is very limited. We believe that the reason the error stays about constant for a small number of measurements (up to 50) is due to the good initial condition that is adopted in these simulations (see below for details on the estimation of the initial condition). For randomly chosen initial conditions, the LASSO-Kalman smoother takes a longer time, and thus requires more observations, to converge.Figure 3 shows a ten-gene directed time-varying network over five time points Figure 3a. For each time point, we assume that seven observations are available. The thickness of the edge indicates the strength of the interaction. Blue edges indicate stimulative interactions, whereas red edges indicate repressive or inhibitive interactions. In order to show the importance of the LASSO formulation and the smoothing, we track the network using the classical Kalman filter Figure 3d, the LASSO online Kalman filter Figure 3c, and the LASSO Kalman smoother Figure 3b. It can be seen that the LASSO constraint is essential in imposing the sparsity of the network, hence significantly reducing the false positive rate. The smoothing improves the estimation accuracy by reducing the variance of the estimate.

In order to obtain a more meaningful statistical evaluation of the proposed LASSO-Kalman, we randomly generated 10,000 sparse ten-gene networks evolving over five time points. The true-positive (TP), true-negative (TN), false-positive (FP), and false-negative (FN) rates, and the sensitivity, specificity, accuracy, and precision are shown in Table 1. The results reported in Table 1 do not take into account the sign or strength of the interactions, but consider only the presence or absence of an interaction between two genes. Observe that the TP rate of the classical Kalman filter is high because the Kalman filter is very dense and contains many spurious connections. This leads to an ‘artificially’ high sensitivity (97% ability to detect edges) but a very low specificity (50% ability to detect the absence of an interaction or sparsity) for the Kalman filter. The smoothed LASSO-Kalman results in a sparser network, missing more edges than the unsmoothed LASSO-Kalman. In particular, the FP rate of the smoothed LASSO-Kalman is higher than its unsmoothed counterpart, but the FN rate of the smoothed LASSO-Kalman is lower, resulting in less spurious connections.

**Table 1:** Performance analysis of the smoothed LASSO-Kalman, unsmoothed LASSO-Kalman, and the classical Kalman filter

	**TP (%)**	**TN (%)**	**FP (%)**	**FN (%)**	**Sensitivity**	**Specificity**	**Accuracy**	**Precision**
Classical Kalman	71.06	13.60	13.11	2.22	0.97	0.50	0.85	0.84
Unsmoothed LASSO-Kalman	80.21	11.52	4.32	3.93	0.95	0.72	0.91	0.94
Smoothed LASSO-Kalman	81.11	10.21	5.63	3.02	0.96	0.64	0.91	0.93

#### 4.1.1 Estimation of *λ*

Equation 14 introduces the penalty parameter *λ*. This parameter controls the sparsity of the resulting estimate, and hence, a correct estimate of *λ* is of paramount importance. Tibshirani [43] enumerates three methods for the estimation of the sparsity parameter: cross-validation, generalized cross-validation, and an analytical unbiased estimate of risk. The first two methods assume that the observations (*X*, *Y*) are drawn from some unknown distribution, and the third method applies to the *X*-fixed case. We adopt the second approach with a slight variation to improve the estimation accuracy. As proposed in [43], this method is based on a linear approximation of the LASSO estimate by the ridge regression estimator. In this paper, instead of calculating the ridge regression estimate as an approximation to the LASSO, we calculate the actual LASSO and determine the number of its effective parameters in order to construct the generalized cross-validation style statistic. The sparsity of the constrained solution is directly proportional to the value of *λ*. If *λ* is small, the solution will be less sparse and if it is large, the solution will be very sparse. At the limit, when *λ*→*∞*, the solution to (14) is the zero vector. To find the optimum value for *λ* for the specific data at hand, we compute the generalized cross-validation statistic for different values of *λ* with a coarse step size to determine the neighborhood of the optimum value of *λ*. Then, we perform a finer search in this neighborhood to find the optimal *λ* for the data. This two-step procedure finds an accurate estimate of *λ* while keeping the computational cost low.

#### 4.1.2 Estimation of the initial condition

The fact that very few observations are available (at each time point) implies that the Kalman filter may take considerable time to converge to the true solution. To make the tracker converge faster, we generate an initial condition based on the maximum likelihood estimate of the static network, as proposed in [11]. This gives the Kalman filter the ability to start from an educated guess of the initial state estimate, which will increase the convergence time of the filter and hence its estimation accuracy over time.

### 4.2 Time-varying gene networks in *Drosophila melanogaster*

A genome-wide microarray profiling of the life cycle of the *D. melanogaster* revealed the evolving nature of the gene expression patterns during the time course of its development [50]. In this study, cDNA microarrays were used to analyze the RNA expression levels of 4,028 genes in wild-type flies examined during 66 sequential time periods beginning at fertilization and spanning embryonic, larval, pupal, and the first 30 days of adulthood. Since early embryos change rapidly, overlapping 1-h periods were sampled; the adults were sampled at multiday intervals [50]. The time points span the embryonic (samples 1 to 30; time E01h until E2324h), larval (samples 31 to 40; time L24h until L105h), pupal (samples 41 to 58; M0h until M96h), and adulthood (samples 59 to 66; A024h until A30d) periods of the organism.

Costello et al. [51] normalized the Arbeitman et al. raw data [50] using the optimized local intensity-dependent normalization (OLIN) algorithm [52]. Details of the normalization protocol can be found at http://www.sciencemag.org/content/suppl/2002/09/26/297.5590.2270.DC1/ArbeitmanSOM.pdf. In their procedure, a gene may be flagged for several reasons: the corresponding transcript not being expressed under the considered condition, the amplification of the printed cDNA was reported as ‘failed’ in the original data, or the data is missing for technical reasons. A statistical test was also conducted to determine if the expression of a labeled sample is significantly above the distribution of background values. Spots with a corrected *p* value greater than 0.01 were considered absent (or within the distribution of background noise). In this study, we downloaded the Costello et al. dataset [51] and considered the unflagged genes only, which amount to a total of 1,863 genes.

**Figure 4:**
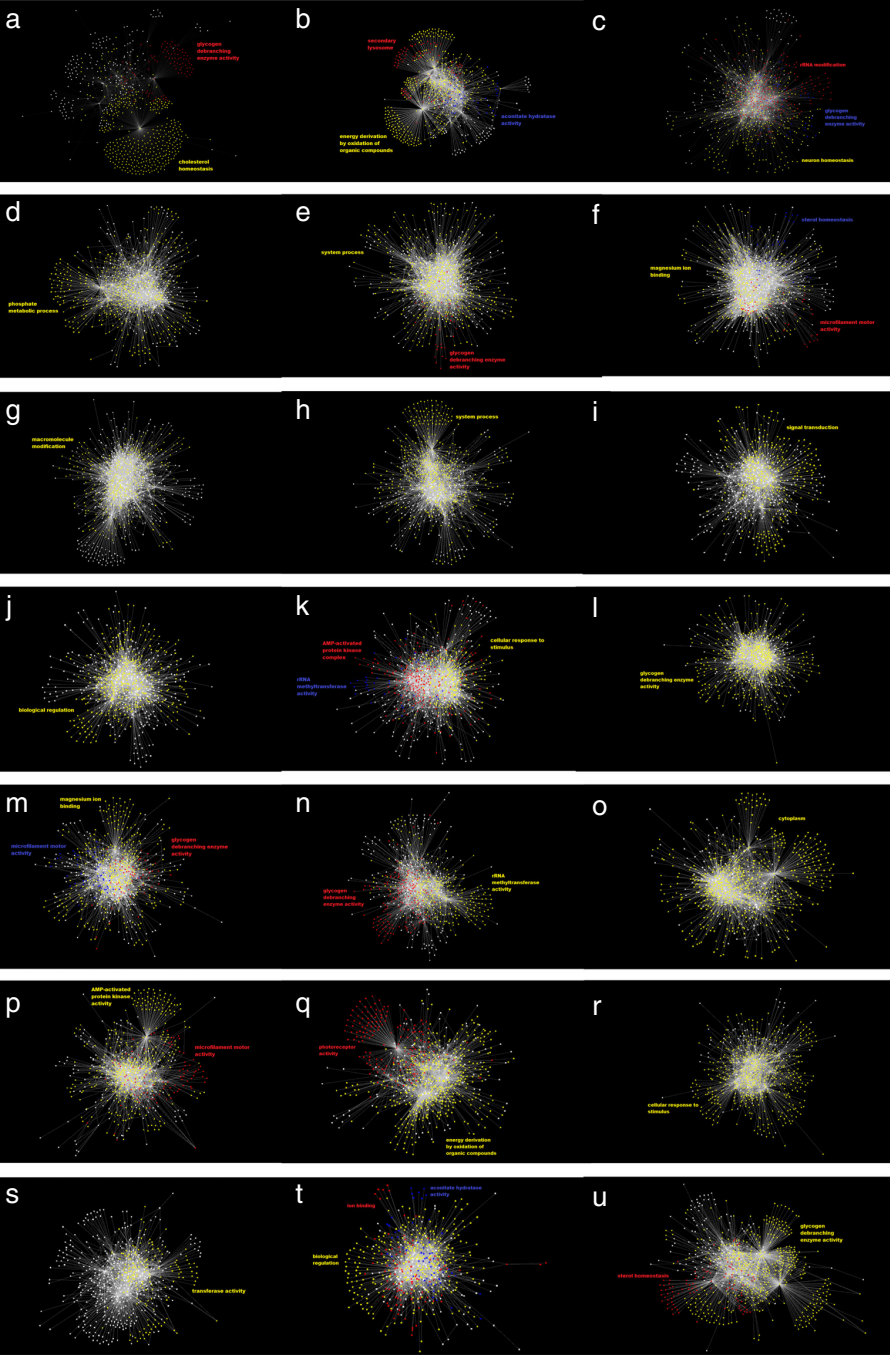
**Snapshots of the time-varying networks at 21 time epochs during the *Drosophila melanogaster * development cycle.** The genes are represented as nodes and interactions as edges. Colored nodes are sets of genes enriched for Gene Ontology summarized by the indicated terms. The nodes were distributed using a force-directed layout in Cytoscape. **(a)***t*_1_ to *t*_3_ (embryonic), **(b)***t*_4_ to *t*_6_ (embryonic), **(c)***t*_7_ to *t*_9_ (embryonic), **(d)***t*_10_ to *t*_12_ (embryonic), **(e)***t*_13_ to *t*_15_ (embryonic), **(f)***t*_16_ to *t*_18_ (embryonic), **(g)***t*_19_ to *t*_21_ (embryonic), **(h)***t*_22_ to *t*_24_ (embryonic), **(i)***t*_25_ to *t*_27_ (embryonic), **(j)***t*_28_ to *t*_30_ (embryonic), **(k)***t*_31_ to *t*_33_ (larval), **(l)***t*_34_ to *t*_36_ (larval), **(m)***t*_37_ to *t*_39_ (larval), **(n)***t*_40_ to *t*_42_ (pupal), **(o)***t*_43_ to *t*_45_ (pupal), **(p)***t*_46_ to *t*_48_ (pupal), **(q)***t*_49_ to *t*_51_ (pupal), **(r)***t*_52_ to *t*_54_ (pupal), **(s)***t*_55_ to *t*_57_ (pupal), **(t)***t*_58_ to *t*_60_ (adult), **(u)***t*_61_ to *t*_63_ (adult).

**Figure 5:**
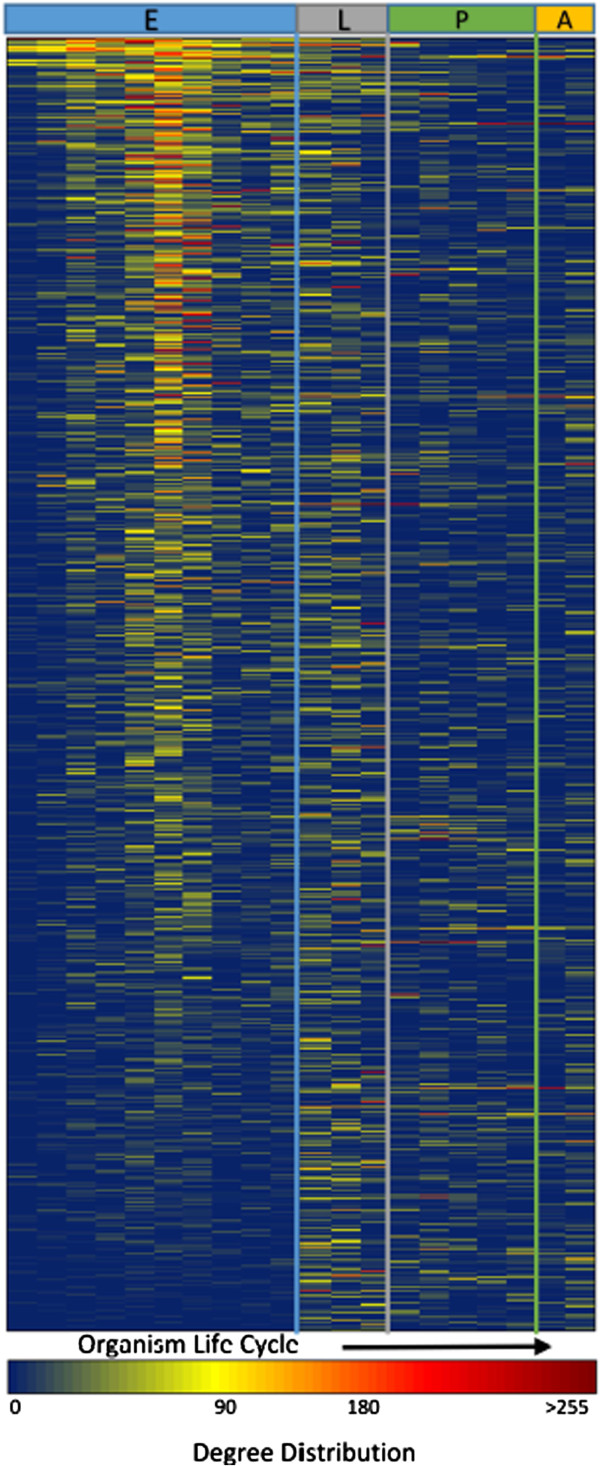
**Gene degree connectivity ordered by the onset of their first increase.** Each row represents data for one gene, and each column is a developmental time point. Blue indicates low degrees, and red indicates high degrees.

The LASSO-Kalman smoother was used to estimate 21 dynamic gene networks, one per three time points, during the life cycle of *D. melanogaster*. Figure 4 shows the estimated networks, where edges with absolute strength less than 10^-3^ were set to zero. The networks were visualized in Cytoscape using a force-directed layout [53]. Markov clustering [54] was used to identify clusters within each network. Clusters containing more than 30 genes were tested for functional enrichment using the BiNGO plugin for Cytoscape [55]. The Gene Ontology term with the highest enrichment in a particular cluster was used to label the cluster on the network. The changing connectivity patterns are an evident indication of the evolution of gene connectivity over time.Figure 5 shows the evolution of the degree connectivity of each gene as a function of time. This plot helps visualize the hubs (high degree nodes) at each time point and shows which genes are active during the phases of the organism’s development. It is clear that certain genes are mainly active during specific developmental phases (transient genes), whereas others seem to play a role during the entire developmental process (permanent genes).

**Figure 6:**
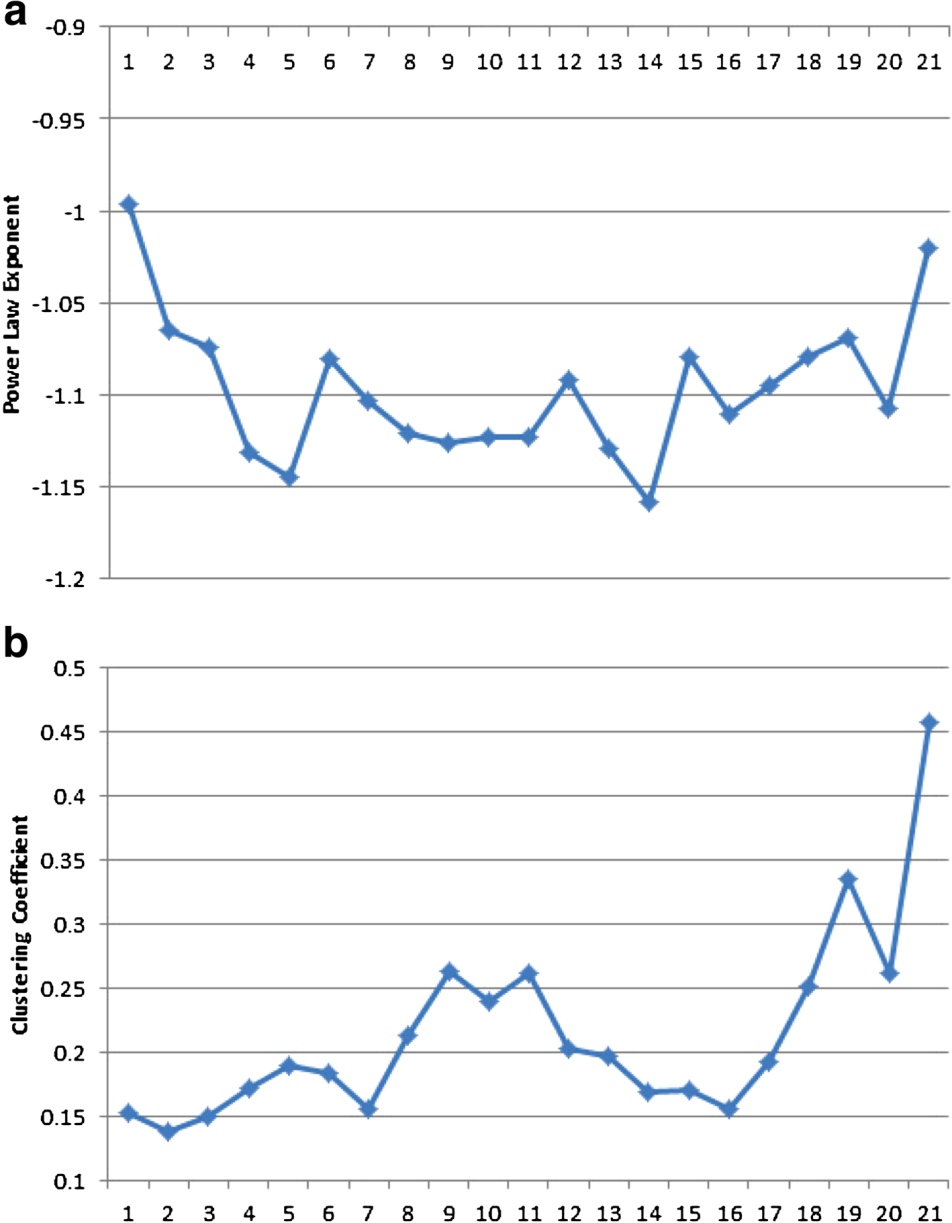
**Temporal network characteristics.(a)** Evolution of the degree distribution using its power law exponent. **(b)** Evolution of the clustering coefficients for each snapshot of the temporal network.

We quantified the structural properties of the temporal network by its degree distribution and clustering coefficient. We found that the degree distribution of each snapshot network follows a power law distribution, which indicates that the networks self-organize into a scale-free state (a global property). The power law exponents of the snapshot networks are plotted in Figure 6a. The clustering coefficient, shown in Figure 6b, measures the cliquishness of a typical neighborhood (a local property) or the degree of coherence inside potential functional modules. Interestingly, the trends (maximums and minimums) of the degree distribution and the clustering coefficients over time corroborate the results in [56], except for the clustering coefficient during early embryonic period. The LASSO-Kalman found a small clustering coefficient in early embryonic, whereas the model-based Tesla algorithm in [56] reported a high clustering coefficient for that phase.

To show the advantages of dynamic networks over a static network, we compared the recovered interactions against a list of known undirected gene interactions hosted in FlyBase (http://flybase.org/). The LASSO-Kalman algorithm was able to recover 1,065 gene interactions (ignoring all interactions smaller or equal than 10^-3^). The static network, computed as one network across all time periods using the algorithm in [11], recovers 248 interactions. Using the segmentation approach, we also computed four networks, where each network uses the number of samples in each developmental phase of the organism (embryonic, larval, pupal, and adulthood). The embryonic-stage network uses the 30 time points sampled during the embryonic phase and recovers 121 interactions. The larval-stage network uses nine time points available for the larval phase and recovers 28 known interactions. The pupal-stage network uses 18 time points collected during the pupal period and recovers 125 interactions. The adult-stage network utilizes eight time points sampled during adulthood and recovers 41 interactions. Hence, in total, the segmentation approach recovers 315 interactions. The dynamic networks of Tesla [56] were able to recover 96 known interactions. We mention that, in [56], the network size was 4,028 genes, whereas we considered a subset of 1,863 unflagged genes. Thus, Tesla’s recovery rate is 2.4*%*, whereas the LASSO-Kalman recovery rate is 57.2*%*. The low recovery rate of Tesla in [56] may be due to the presence of spurious samples since the flagged genes were included in the networks.

### 4.3 High-performance computing implementation

**Figure 7:**
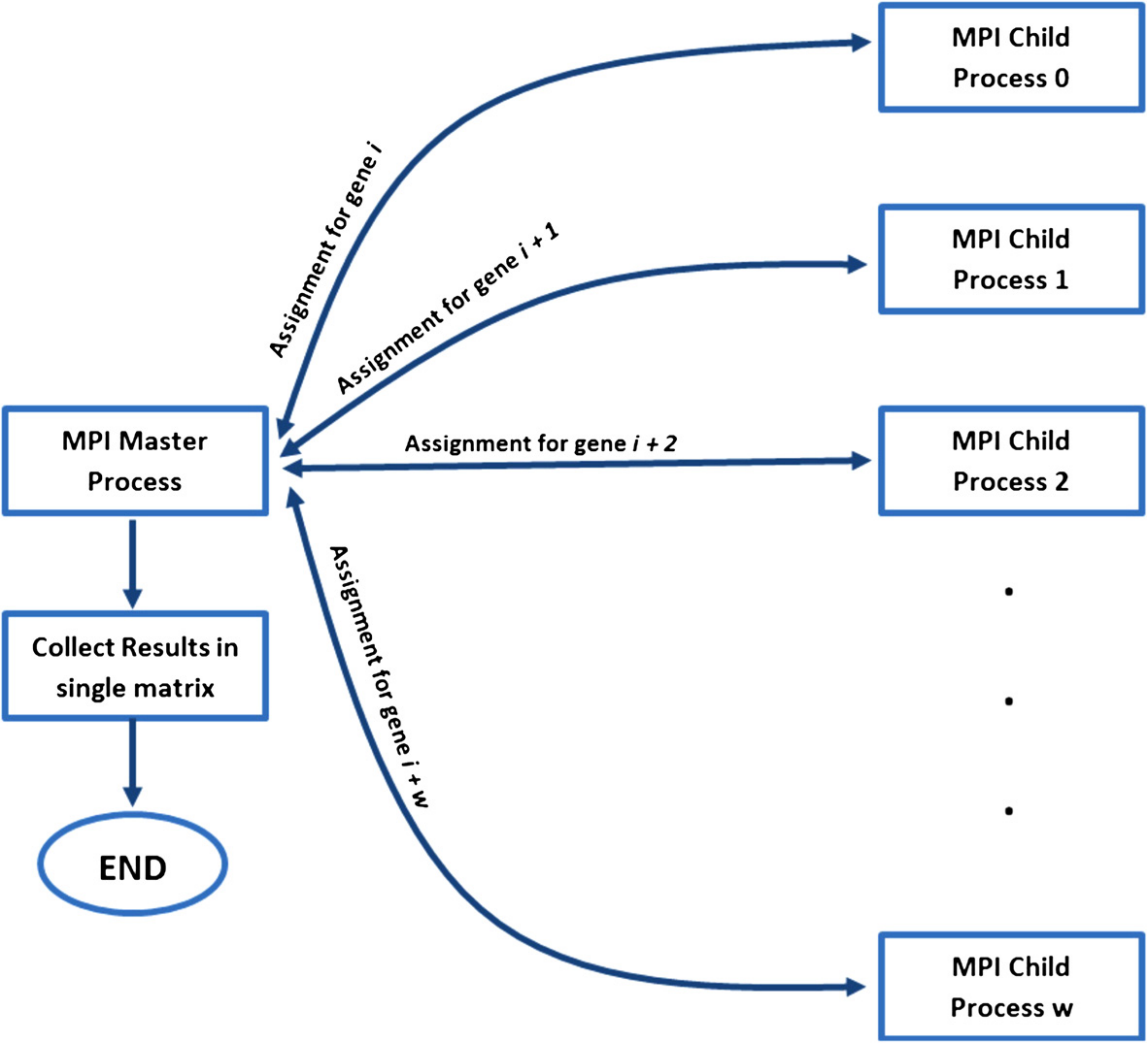
High-performance computing implementation.

The proposed LASSO-Kalman smoother algorithm was first tested and validated in MATLAB. Subsequently, a high-performance computing (HPC)-based implementation of the algorithm was developed to allow a large number of genes. Each HPC core computes the interactions of one gene at a time. The communication between the individual processes is coordinated by the open message passing interface (open MPI). Due to the large scale of the problem, both the Intel®; C++ Compiler and the Intel®; Math Kernel Library (Intel®; MKL) (Intel Corporation, Santa Clara, CA, USA) were used on a Linux-based platform for maximum performance. This approach enabled an implementation that is highly efficient, inherently parallel, and has built-in support for the HPC architecture. The implementation starts by the main MPI process spawning the child processes: each child process is assigned an individual gene to compute, based on the gene expression data that is made available to it using the file system. The child process returns the computed result to the main process, which then assigns the next gene until all genes are processed. Finally, the master process compiles the computed results in a contagious matrix. Figure 7 summarizes the HPC implementation process. The memory requirement of the algorithm, however, is still high. At each time point, two *p*×*p* covariance matrices must be stored and computed (the *a priori* and *a posteriori* error covariance matrices), where *p* is the number of genes. In order to alleviate the memory requirement, we used a memory mapped file, which swaps the data between the local disk and the memory. We used the Razor II HPC system at the Arkansas High Performance Computing Center (AHPCC) at the University of Arkansas at Fayetteville. The AHPCC has 16 cores per node, with 32 GB of memory; each node is interconnected using a 40-Gbps QLogic quad-data rate QDR InfiniBand (Aliso Viejo, CA, USA). In our implementation, we were allowed to use 40 such nodes at a given time. This implementation is scalable and supports a larger number of genes for future investigations. Further details of the implementation are available at http://users.rowan.edu/~bouaynaya/EURASIP2014.

## 5 Conclusions

Due to the dynamic nature of biological processes, biological networks undergo systematic rewiring in response to cellular requirements and environmental changes. These changes in network topology are imperceptible when estimating a static ‘average’ network for all time points. The dynamic view of genetic regulatory networks reveals the temporal information about the onset and duration of genetic interactions, in particular showing that few genes are permanent players in the cellular function while others act transiently during certain phases or ‘regimes’ of the biological process. It is, therefore, essential to develop methods that capture the temporal evolution of genetic networks and allow the study of phase-specific genetic regulation and the prediction of network structures under given cellular and environmental conditions.

In this paper, we formulated the reverse-engineering of time-varying networks, from a limited number of observations, as a tracking problem in a compressed domain. Under the assumption of linear dynamics, we derived the LASSO-Kalman smoother, which provides the optimal minimum mean-square sparse estimate of the connectivity structure. The estimated networks reveal that genetic interactions undergo significant rewiring during the developmental process of an organism such as the *D. melanogaster*. We anticipate that these topological changes and phase-specific interactions apply to other genetic networks underlying dynamic biological processes, such as cancer progression and therapeutic treatment and development.

Finally, we anticipate that the rapid breakthroughs in genomic technologies for measurement and data collection will make the static representation of biological networks obsolete and establish instead the dynamic perspective of biological interactions.

## Competing interests

The authors declare that they have no competing interests.
